# Moral Decision-Making During COVID-19: Moral Judgements, Moralisation, and Everyday Behaviour

**DOI:** 10.3389/fpsyg.2021.769177

**Published:** 2022-02-04

**Authors:** Kathryn B. Francis, Carolyn B. McNabb

**Affiliations:** ^1^School of Psychology, Keele University, Keele, United Kingdom; ^2^Department of Psychology, University of Bradford, Bradford, United Kingdom; ^3^School of Psychology and Clinical Language Sciences, University of Reading, Reading, United Kingdom

**Keywords:** COVID-19, moral decision-making, moralisation, utilitarian, behaviour

## Abstract

The COVID-19 pandemic continues to pose significant health, economic, and social challenges. Given that many of these challenges have moral relevance, the present studies investigate whether the COVID-19 pandemic is influencing moral decision-making and whether moralisation of behaviours specific to the crisis predict adherence to government-recommended behaviours. Whilst we find no evidence that utilitarian endorsements have changed during the pandemic at two separate timepoints, individuals have moralised non-compliant behaviours associated with the pandemic such as failing to physically distance themselves from others. Importantly, our findings show that this moralisation predicts sustained individual compliance with government-recommended behaviours.

## Introduction

By November 2021, the novel coronavirus disease (COVID-19) had infected more than 249 million people worldwide (WHO, 2021). COVID-19 poses significant global challenges, having drastic health, social, and economic impacts (e.g., Anderson et al., 2020; McKibbin and Fernando, 2020; Sohrabi et al., 2020). Within the behavioural sciences, research into COVID-19 has been motivated by attempts to “nudge” public behaviours in-line with government recommendations (e.g., social distancing; Jordan et al., 2021; Lunn et al., 2020).

At present, there is only limited research investigating moral decision-making in the time of COVID-19. This is an important area of research as significant global changes in social structures and community practises are likely to affect people’s judgements about what is now “right” and “wrong” as well as the moral principles that guide their decision-making. Several features of the pandemic have moral overtones that are relevant to moral decision-making research. Firstly, the pandemic requires that people make sacrifices for the wellbeing of others (or *for the greater good*) and secondly, the current situation has introduced new social (or moral) norms such as physically distancing from others. As an example, the United Kingdom public have been regularly reminded to “Stay Home, Protect the NHS, Save Lives.”

Traditionally, moral decision-making is investigated using sacrificial moral dilemmas originally adopted from philosophy. In a well-known example, the *footbridge* dilemma, participants are asked whether they would push a very large stranger off a bridge into the path of a runaway trolley in order to stop it from hitting and killing five other people (Foot, 1978). Responses to these dilemmas supposedly represent the tension between two moral ideals or schools of moral thought: “characteristically” utilitarian (i.e., approving of sacrificing one life to save several others) and “characteristically” deontological (i.e., disapproving of sacrificing one life to save several) (Greene, 2009).

But do preferences for these characteristically utilitarian or deontological schools of thought guide related judgements and behaviours in real-life? Little research has investigated the extent to which people’s moral decisions in sacrificial dilemma or their utilitarian preferences predict their judgements and actions in a real-world conflict or dilemma. Of course, sacrificial moral dilemmas were not created with the intention of predicting real-world decisions, but to allow moral conflicts to arise in artificial contexts with anonymous agents (Christensen and Gomila, 2012). However, there have been attempts to explicate moral action from these measures. For example, attempts have been made to increase their contextual saliency through using virtual reality (Navarrete et al., 2012; Francis et al., 2016).

Crucially, sacrificial moral dilemmas only capture a single component of utilitarianism; namely *instrumental harm* or whether to sacrifice one life to save many (Everett et al., 2018). Of equal importance, is the positive dimension of utilitarianism or *impartial beneficence*, which posits that we must promote the greater good for all sentient life in an impartial way (Singer, 1979). Recently, Kahane et al. (2018) proposed a two-dimensional model of utilitarianism and corresponding measure, to assess both instrumental harm *and* impartial beneficence in order to address existing limitations of sacrificial moral dilemmas. Importantly, little research has attempted to determine whether utilitarian endorsements (whether positive or negative) assessed via these measures, can be applied to real-world moral conflicts to predict real-world decisions.

The COVID-19 pandemic is one example of a real-world crisis that presents a number of distressing and ethical challenges and thus, provides a unique opportunity for such an investigation. In fact, recent research has found that individual differences in moral intuitions (such as caring and fairness) predict behaviour compliance versus resistance during the pandemic (Chan, 2021). However, research has yet to investigate whether individual differences in endorsements of impartial beneficence and/or instrumental harm predict behaviour compliance versus resistance during the pandemic. It is also important to consider how endorsing these principles would affect moral decisions pertaining to the pandemic. For example, one utilitarian decision consistent with instrumental harm might be to remove government restrictions, causing harm to elderly populations, in order to bring about greater good (social and economic benefits) for younger populations. However, a contrasting utilitarian decision consistent with impartial beneficence, would be to value all life, and thus endorse government restrictions to reduce *all* deaths regardless of age. Thus, it is important to conceptualise and measure utilitarianism according to both positive and negative dimensions.

While people may generally adhere to one school of moral thought or another, there is evidence to suggest that features of the situation and characteristics of the decision-maker can influence or change related moral judgements. For example, researchers find that exposing individuals to moral dilemmas framed positively (in terms of “lives saved”) elicits more utilitarian responses than those framed negatively (in terms of “lives lost”) (Cao et al., 2017; McDonald et al., 2021). The implications of the above research go beyond abstract theory as this suggests that the way in which individuals receive information in a given context, is likely to influence their decisions. During the COVID-19 pandemic for example, emphasis has been placed on following regulations to *protect ourselves and our community’s health* (Chan, 2021) and how these messages are framed to members of the public during the pandemic could influence their moral decisions.

Additionally, the extent to which individuals assign moral value to an issue can influence judgements and behaviours (e.g., Mulder et al., 2015). When a once neutral issue is assigned moral value (through the process of moralisation), it gains emotional and motivational salience (e.g., Rozin, 1999). This moralisation can shape social norms around certain behaviours such as adhering to government behavioural guidelines. The moralisation of smoking, for example, has significantly predicted a decline in smoking behaviours (Rock et al., 2007). When individuals no longer imbue an issue with moral values, de-moralisation occurs, and the same issue can then be imbued with moral value again through a process of re-moralisation. Of course, moralisation can also produce reactive responses. For example, the moralisation of obesity and meat consumption often results in individuals feeling morally judged and subsequently refusing to adhere to healthier diets or vegetarianism (e.g., Minson and Monin, 2012). As such, moralisation is a dynamic and fluid process affected by context and time. With regards to the COVID-19 pandemic, existing research has found evidence that eliminating the virus has become moralised to the extent that individuals evaluate harmful outcomes as more tolerable if they resulted from attempts to eliminate COVID-19 (Graso et al., 2021). This would suggest that moralisation of behaviours associated with the pandemic has shaped social norms around compliant behaviours although this has yet to be empirically investigated.

In terms of consistency in moral decision-making, there is limited existing research investigating moralisation and moral judgement over time *and* context. Of the research in this area, there is some evidence that individual moral judgements are consistent across time and are thus anchored to moral principles (Helzer et al., 2017). However, research has yet to consider how significant changes to an individual’s environment may affect moral judgements and underlying principles over time. Given the contextual changes arising from the pandemic mentioned previously, COVID-19 provides a unique opportunity to investigate utilitarian preferences across time and in a rapidly changing context.

In this study, we investigate whether utilitarian preferences predict compliance to government recommendations during the pandemic and whether utilitarian preferences change during the pandemic. While sacrificial moral dilemmas are characteristically improbable, how responses to them are affected by external experiences like the pandemic, is not well understood. To account for the positive dimension of utilitarianism, we consider how both instrumental harm *and* impartial beneficence are affected by time and context. To account for moralisation, we also assess the extent to which everyday behaviours associated with the COVID-19 pandemic (such as failing to physically distance) have become moralised and whether assigning moral values to these behaviours predicts adherence to government recommendations.

A significant strength of the present study is in its longitudinal design. In August 2019, we conducted a study to investigate the within-person consistency of moral decision-making across a range of hypothetical scenarios (timepoint 1). Since that time, the COVID-19 pandemic has provided a rare opportunity to investigate the psychological impacts of a sudden-onset global crisis in the general population. Any impact of such an event on moral decision-making, could signify a change in the way people evaluate moral dilemmas or indicate a shift in their utilitarian preferences. Additionally, if responses to utilitarian measures change (become more or less utilitarian) over time or are able to predict behaviour (i.e., conforming to government-recommended guidelines on social distancing and hygiene), this would suggest that they are associated with related decisions in the real-world. Data were collected at two timepoints during the pandemic (timepoint 2: April 2020; and timepoint 3: September/October 2020) to ensure reliability of results. Experimental materials, pre-registration, data, analysis code, and the results from this study are available on the OSF: https://osf.io/u5a3t/.

## Research Questions

Question (I): Do utilitarian preferences prior to and during the COVID-19 pandemic predict compliance with government-recommendations? Research has found that individual differences in moral intuitions (such as caring and fairness) predict behaviour compliance versus resistance during the pandemic (Chan, 2021). However, research has yet to investigate whether individual differences in endorsements of impartial beneficence and/or instrumental harm predict behaviour compliance during the pandemic. Given that endorsing instrumental harm versus impartial beneficence could result in different endorsements of utilitarian decisions pertaining to the pandemic (see previous examples), the positive and negative dimensions of utilitarianism and their relationship to compliance are assessed separately.

Question (II): Will moral decision-making differ before versus during the COVID-19 pandemic? Individuals are being regularly exposed to public messages and contexts that ask that they act responsibly to help others. Exposure to these contexts may result in a positive framing effect (focussing on saving lives) subsequently increasing utilitarianism (Cao et al., 2017) compared to pre-pandemic responses. There is some evidence that increased focus on our own mortality results in individuals becoming less utilitarian (Tremoliere et al., 2012). A recent study found that people display less preference for utilitarian judgements in moral dilemmas during the pandemic and they explain these findings with reference to mortality salience (Antoniou et al., 2021). However, this mortality salience effect has failed to replicate (Klein et al., 2019).

Question (III): Does assigning moral values to (moralising) current pandemic-related behaviours predict engagement in government-recommended behaviours? It is likely that moralisation will shape social norms in favour of adhering to these recommendations given recent evidence that elimination of COVID-19 has become moralised (Graso et al., 2021). However, it is also important to acknowledge that moralisation may result in individuals feeling judged and subsequently decreasing their adherence to these behaviours (e.g., Mulder et al., 2015).

## Materials and Methods

### Participants

In order to capitalise on the strengths of a repeated-measures design (especially valuable in the context of a long-term pandemic event), we invited participants from a study conducted prior to the pandemic (baseline), *N* = 107^1^, to take part in the current (COVID-19) study. This limited our potential pool of participants; however, we believe this longitudinal design enabled us to make inferences about how the pandemic has affected decision-making *within* individuals over time and subsequently, adds value to the field. Importantly, the opportunistic nature of this study does constrain us to the measures used to evaluate moral decision-making in the original study (completed before the pandemic). Although our sample size is limited, the current study has 80% power to detect a medium within-participant effect of *f* ≥ 0.367 across the three timepoints [sensitivity analysis conducted with WebPower_0.5.2 in R version 4.0.2 (2020-06-22); formula: wp.rmanova (*n* = 73, ng = 1, nm = 3, alpha = 0.05, power = 0.8, type = 1, *f* = NULL)] and a medium effect size of *r* ≥ 0.302 for the correlation between baseline moral preferences (sacrificial dilemmas and OUS)/moralisation of everyday behaviours and behaviour compliance during the pandemic [formula: wp.correlation (*n* = 83, power = 0.8, alpha = 0.05, *r* = NULL)].

**FIGURE 1:**
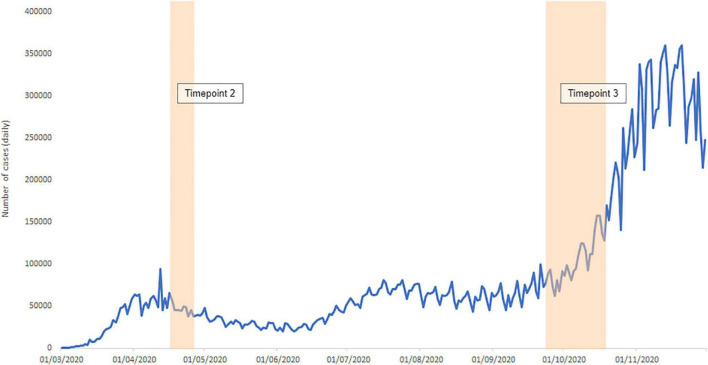
Timeline showing data collection intervals during the pandemic in the context of daily case rate (sum across sample countries-of-residence). Vertical shaded blocks represent data collection periods in 17th–26th April 2020 (timepoint 2) and 25th September–19th October 2020 (timepoint 3). Note that at timepoint 2, national or localised lockdowns were in place in the United Kingdom (60.2% of sample) and the majority of other countries-of-residence sampled. Two participants were resident in Hungary where localised recommendations were in place rather than lockdowns. At timepoint 3, all countries sampled were not in national lockdowns. COVID-19 data taken from COVID-19 Data Repository by the Center for Systems Science and Engineering (CSSE) at Johns Hopkins University. Cases are daily new confirmed cases of COVID-19 summed across the countries-of-residence sampled.

In total, 83 participants (47.0% female, *M*_age_ = 35.5 years, *SD*_age*e*_ = 12.6 years, 60.2% living in the United Kingdom) of the original 107 completed the COVID-19 study at timepoint 2 (April 2020 during national/localised lockdowns in the United Kingdom and the majority of other countries of residence^2^) and 73 participants (46.6% female, *M*_age_ = 36.32 years, *SD*_age_ = 13.00 years, 60.3% living in the United Kingdom) of those who took part at timepoint 2 also completed the COVID-19 study at timepoint 3 (September–October 2020 when national lockdowns were no longer in place in the countries sampled) (see Figure 1). One participant did not disclose their gender. In both studies, all participants were at least 18 years of age. There were no gender, race, or religious constraints on study participation. All participants had good (or corrected) vision, basic literacy skills, and proficiency in English. Participants were invited to participate through Prolific and only those with a high Approval Rate (>87) were permitted to enrol. Participants were paid £5.02/h for participation in the original study (timepoint 1) plus £7.50/h for each COVID-19 study (timepoint 2, timepoint 3). Both studies received ethical approval from respective committees at the University of Bradford (2019-E747; 2020-E803) and the University of Reading (2019-013-CM; 2020-044-CM) and all participants gave informed consent.

### Measures

#### Moral Measures (Longitudinal Analyses)

In the baseline study (timepoint 1) and COVID-19 studies (timepoint 2, timepoint 3), participants responded to 14 sacrificial moral dilemmas presented in randomised order for each participant. Moral dilemmas used in the study are provided in detail in the online Supplementary Material and OSF page^3^ but scenarios were formatted as follows:

*A runaway trolley is speeding down the tracks toward five workmen who will be killed if the trolley continues on its present course. You are standing next to the tracks, but you are too far away to warn them. Next to you, there is a very large stranger.*

*If you push the large stranger onto the tracks, the trolley will slide off the tracks and won’t continue its course toward the workmen. This will kill the stranger, but you will save the five workmen.*

*Do you cause the trolley to derail by pushing the stranger onto the tracks, so the trolley does not reach the five workmen?*

After reading a dilemma, participants were asked whether they would perform the action described (sacrificing one life to save another). They responded by selecting “Yes” or “No” with a “Yes” response being characteristically utilitarian. Sacrificial moral dilemmas assess instrumental harm or permissive attitudes toward sacrificing one life to save many more.

**FIGURE 2:**
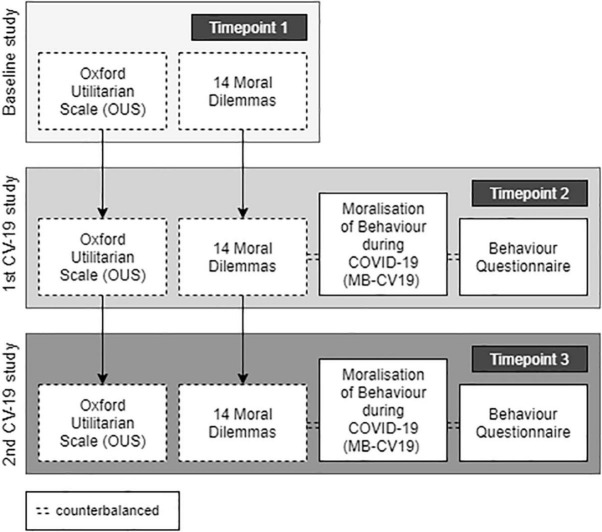
Experimental designs of baseline and COVID-19 studies. Participants who took part in the COVID-19 studies (timepoint 2, timepoint 3) had previously taken part in a baseline study (timepoint 1). Participants responded to the OUS and moral dilemmas at all timepoints (arrows indicate longitudinal comparison).

Given that sacrificial moral dilemmas do not allow assessment of impartial beneficence (promoting the greater good for all sentient life in an impartial way) and to account for the multidimensional nature of utilitarianism, we also measured utilitarian tendencies (both instrumental harm and impartial beneficence), using the *Oxford Utilitarianism Scale* (OUS; Kahane et al., 2018). The OUS contains two subscales to measure the positive dimension of utilitarianism (impartial concern for the greater good or *Impartial Beneficence*; five items; α_baseline_ = 0.72; α_COVID–19_ = 0.73) and the negative dimension (permissive attitude toward *Instrumental Harm*; four items; α_baseline_ = 0.73; α_COVID–19_ = 0.61). The scale contains nine items in total, rated on a 7-point Likert-type scale (1 = not at all wrong, 7 = extremely wrong), including items such as “It is just as wrong to fail to help someone as it is to actively harm them yourself.” Participants completed the OUS at all timepoints (during the baseline and COVID-19 studies; see Figure 2).

#### COVID-19 Specific Measures of Morality and Behaviour

To measure moralisation of behaviours during the COVID-19 pandemic, we adapted existing items from the *Moralisation of Everyday Life Scale* (MELS; Lovett et al., 2012) to create a *Moralisation of Behaviour during COVID-19 Scale* (MB-CV19) which includes violations of social distancing behaviours, personal hygiene, volunteering behaviours, and endorsements of hoarding behaviours (20 items; α = 0.89; see Supplementary Material). Participants were asked “*How much do you consider this behaviour to be morally wrong*?” and responded on a 7-point Likert-type scale (0 = not wrong at all; a perfectly OK action, 6 = very wrong; an extremely immoral action). A principal components analysis (PCA) was conducted for MB-CV19 in IBM SPSS (version 26) with components analysed in R. According to Kaiser’s criterion, PCA of the MB-CV19 produced five components. As most items loaded onto multiple components, a mean overall score was used in the main analysis.

We anticipated that people’s moral principles (measured using sacrificial dilemmas and the OUS) as well as how much they moralised previously neutral behaviours, would impact their adherence to government-recommended protocols. Participants responded to 11 statements about their current behaviours which included social distancing behaviours, personal hygiene behaviours and volunteering behaviours (see Supplementary Material). Participants were asked “*Which of the following behaviours are you engaging in and how often?*” and could respond on a 5-point Likert scale (1 = I never do this, 5 = I always do this). A principal components analysis (PCA) was conducted for the behaviour questionnaire (at baseline) in SPSS with components analysed in R. PCA of current behaviours produced a single component (composite behaviour score; α = 0.85) that was used for all subsequent analyses of behaviour (full details of PCA in Supplementary Material).

### Experimental Procedure and Data Analysis

All tasks were created using the Gorilla platform^4^ and are publicly available^5^. For consistency, the OUS, which was presented first in the baseline study (timepoint 1), was also presented first in the COVID-19 studies (timepoint 2, timepoint 3). Participants then responded to the sacrificial moral dilemmas. In the COVID-19 studies (timepoint 2, timepoint 3), the MB-CV19 and the measure of current behaviours were also presented (Figure 2). Finally, participants were asked to answer two questions related to whether they or someone in their immediate social environment had confirmed or suspected COVID-19.

Statistical analysis was conducted in R and all results cross-validated by both researchers and alternative software (SPSS 26). We report all manipulations, measures, and exclusions in these studies. Analyses were preregistered^6^. Descriptions of statistical analyses for each research question are provided in the section “Results.” Prior to regression analyses (for Question I and Question III), data were assessed for assumptions of linearity, homoscedasticity, normality and influential cases. Data met assumptions for linear regression. Details of all diagnostics can be found in the Supplementary Material. Full analysis pipelines are available on the OSF: https://osf.io/u5a3t/.

In the first COVID-19 study (timepoint 2), 21.7% of participants reported having COVID-19 (themselves or a loved one). In the second COVID-19 study (timepoint 3), this had increased to 26% of participants. As no associations were identified between COVID-19 experience and MB-CV19 or current behaviours at timepoints 2 or 3 (*ps* > 0.419), experience was not included in further analyses (see Supplementary Material). Previous and current self-reported trust in government was collected at timepoint 3, following evidence that this influences compliance with guidelines (Wright et al., 2020; see Supplementary Material). As no associations were identified between these self-reported trust measures and MB-CV19 or current behaviours (*ps* > 0.324), trust was not included in further analyses.

## Results

### Question I: Do Utilitarian Preferences Predict Compliance With Government Recommendations?

To establish whether utilitarian preferences (from sacrificial dilemmas and from the OUS) prior to and during COVID-19, predict behaviours during the pandemic, we conducted simple linear regressions with utilitarian proportion and OUS score (total and subscale scores) at baseline against composite behaviour score in the first COVID-19 study (timepoint 2) and second COVID-19 study (timepoint 3). One influential case was identified after assumption tests at both timepoints 2 and 3 and was removed.

**FIGURE 3:**
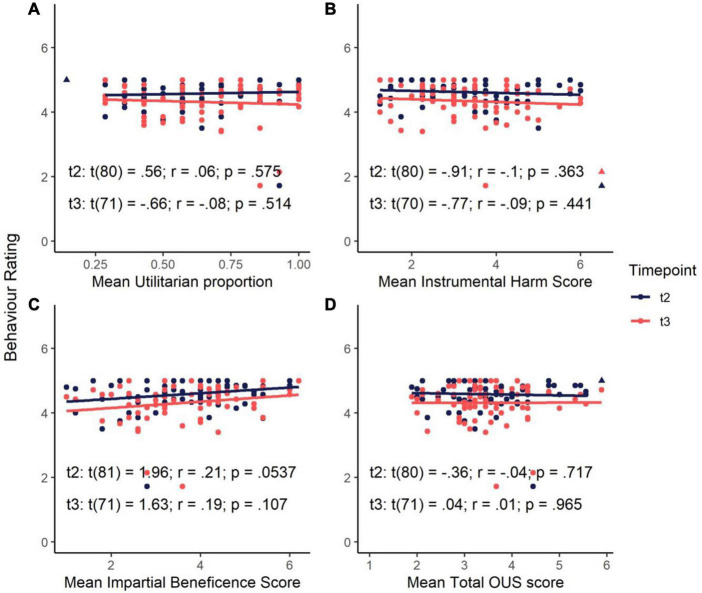
Association between engagement in COVID-19-related behaviours at timepoints 2 and 3 and baseline **(A)** utilitarian proportion in sacrificial moral dilemmas, **(B)** mean Instrumental Harm score, **(C)** mean Impartial Beneficence score and, **(D)** mean total Oxford Utilitarianism Scale (OUS) score. Outliers removed from the regression analyses are marked with triangles.

People’s responses to sacrificial moral dilemmas and their attitudes toward instrumental harm and impartial beneficence did not predict their adherence to government-recommended guidelines related to the COVID-19 pandemic. As mean OUS scores and utilitarian proportions did not differ significantly between baseline (timepoint 1) and the COVID-19 pandemic (timepoints 2 and 3), only baseline data were compared to composite behaviour scores (see preregistration: see text footnote 6). Baseline utilitarian proportions (moral judgements) did not significantly predict behaviour scores at timepoint 2 (*p* = 0.575) or timepoint 3 (*p* = 0.514) nor did overall OUS scores, OUS-IH or OUS-IB scores significantly predict behaviour scores at timepoint 2 (*ps* > 0.054) or timepoint 3 (*ps* > 0.107) (see Figure 3).

### Question II: Has the COVID-19 Pandemic Influenced Utilitarian Preferences?

One-way repeated measures ANOVAs^7^ were used to compare utilitarian proportions (the proportion of utilitarian responses across sacrificial moral dilemmas), and mean OUS overall and subscale scores for each participant before (timepoint 1), during the COVID-19 pandemic in April (timepoint 2) and during the COVID-19 pandemic in September–October (timepoint 3).

**FIGURE 4:**
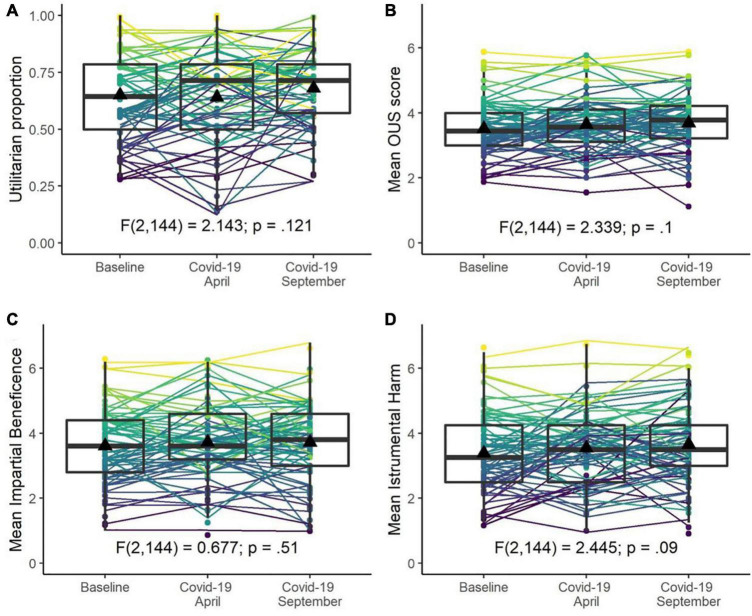
Moral decision-making before and during the COVID-19 pandemic. Proportion utilitarian responses to moral dilemmas **(A)**, mean Oxford Utilitarianism Scale (OUS) score **(B)**, mean Impartial Beneficence subscale score **(C)**, and mean Instrumental Harm subscale score **(D)** at baseline (timepoint 1) and during the COVID-19 pandemic (timepoints 2 (Covid-19 April) and 3 (Covid-19 September). Boxplots show medians and interquartile ranges; triangles represent mean scores for each timepoint. Colours represent baseline scores for each participant.

The COVID-19 pandemic had no statistically significant influence on utilitarian preferences. Utilitarian proportions in sacrificial moral dilemmas were similar prior to the pandemic (timepoint 1: *M* = 0.65, *SD* = 0.21) and during the COVID-19 pandemic at timepoint 2 (*M* = 0.64, *SD* = 0.21) and timepoint 3 (*M* = 0.68, *SD* = 0.17) and were not significantly different (*p* = 0.121) (see Figure 4A). Scores on the OUS (total, IB, IH) prior to the pandemic (timepoint 1) were similar to scores during the pandemic at timepoints 2 and 3 (see Supplementary Table 3) and were not significantly different, (*ps* > 0.090) (see Figures 4B–D).

### Question III: Is Moralisation of Everyday Behaviours During the COVID-19 Pandemic Associated With Compliance With Government Recommendations?

To establish whether moralisation predicted engagement in government-recommended behaviours at both timepoints during the COVID-19 pandemic, we conducted simple linear regressions.

**FIGURE 5:**
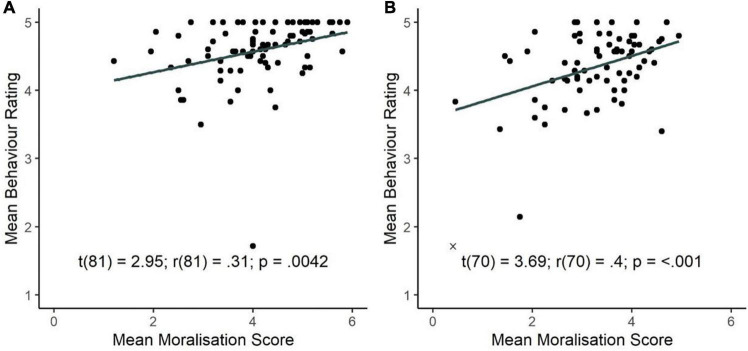
Relationship between moralisation of government-recommendations (mean MB-CV19 score) and engagement in government-recommended behaviours during COVID-19 pandemic at **(A)** timepoint 2 (April 2020) and **(B)** timepoint 3 (September 2020). Excluded participant is denoted by “x” and was not included in the regression.

Mean moralisation scores (measured using the MB-CV19) were positively correlated with engagement in government-recommended behaviours at both timepoints during the pandemic. At timepoint 2 mean moralisation scores positively predicted composite behaviour scores, *r*(82) = 0.31, *p* = 0.004, with the regression model explaining 9.7% of the variance in behaviour scores, *F*(1,81) = 8.68, *p* = 0.004, β = 0.15, 95% CI (0.05, 0.25) (see Figure 5A). This relationship was replicated at timepoint 3 (later during the pandemic), *r*(71) = 0.40, *p* < 0.001, with the regression model explaining 16.3% of the variance in behaviour scores, *F*(1,70) = 13.65, *p* < 0.001, β = 0.22, 95% CI (0.10, 0.34). Participants who moralised everyday pandemic-related behaviours, were more likely to engage in government-recommend behaviours at both timepoint 2 and 3 (see Figure 5B).

## Discussion

The COVID-19 pandemic provides a rare opportunity to investigate the impacts of a global health crisis on people’s moral decision-making and to understand how the moralisation of government recommendations might influence people’s behaviours. We find evidence that behaviours associated with the pandemic are moralised and that this moralisation predicts compliance with government recommended behaviours including social distancing and hygiene. Importantly, this relationship is stable, even under different national and localised restrictions. In the present studies, we do not find evidence that utilitarian preferences predict compliance with government recommendations or that the rapidly changing context of the pandemic has influenced the moral principles that anchor individual moral judgements.

### Question III: Moralisation of Everyday Behaviours During the COVID-19 Pandemic and Compliance With Government Recommendations

In terms of the relationship between moralisation and behaviour, we proposed that assigning moral values to behaviours associated with COVID-19 may shape social norms in favour of adhering to these behaviours (Mulder et al., 2015; Graso et al., 2021). However, and given previous research, we also acknowledged that moralisation could prompt reactance responses and less adherence to these behaviours (e.g., Mulder et al., 2015). Overall, assigning moral value to behaviours during the COVID-19 pandemic predicted self-reported engagement in government-recommendations in April 2020 and again in September 2020. The scores on the MB-CV19 alone suggest that changes to the current context have moved what were previously neutral behaviours (e.g., arranging a house party or visiting your grandparents) into the moral domain (e.g., Brown, 2018). Subsequently, this moralisation may have prompted the development of social norms that motivate individuals to adhere to corresponding behaviours (e.g., Mulder et al., 2015). To determine if moralisation influences behaviour via social norms, future research should assess and/or manipulate social norms directly to determine their role in this process. Importantly, moralisation is sustained across several months with this research demonstrating that these norms were retained even at a time when national and/or localised lockdown restrictions were being eased or had been removed (September–October 2020). This finding has several important implications. Firstly, previous research has found conflicting evidence regarding the effectiveness of moralisation in public messaging (e.g., Stok et al., 2014; Mulder et al., 2015) with people often reacting negatively to moralised messages such as in the case of health-related choices (e.g., Minson and Monin, 2012). However, and in this case, moralising non-compliance with government-recommended or antisocial behaviours may be an effective method in reducing harm. As such, public messaging campaigns that emphasise moralisation could be most effective in encouraging complimentary behaviours (e.g., Cialdini et al., 2006).

### Question II: The Influence of the COVID-19 Pandemic on Utilitarian Preferences

In terms of utilitarian preferences before and during the pandemic, we originally suggested that increased exposure to positive framed messaging campaigns and various contexts promoting the saving of lives, may result in increased utilitarian endorsements during the pandemic (Cao et al., 2017). Our results do not provide support for this as utilitarian proportions in response to sacrificial moral dilemmas, instrumental harm endorsements, and partial beneficence endorsements were consistent over time, remaining unchanged during the pandemic (in both April and September 2020).

One explanation for the discrepancy in our findings and previous research is that previous framing studies employed controlled manipulations in text-based moral dilemmas (e.g., Cao et al., 2017) whereas in the present study, contextual changes relied on exposure to real-world messaging campaigns which we did not have control over. It is also important to highlight that sacrificial moral dilemmas and the OUS were designed to assess trait-level moral preferences. While personal experience during the pandemic is likely to affect states, beliefs at the core of a person’s moral compass may remain unaffected (e.g., Francis et al., 2018). Thus, this research does provide additional support for longitudinal research showing consistency in utilitarian preferences over time and context (Helzer et al., 2017). Despite variability within individuals, utilitarian preferences remained stable over time, and we did not find evidence that these principles change in a global pandemic (see Supplementary Material for similar repeated-measures results). This supports recent findings showing that while people displayed a preference for utilitarian triage decisions during the pandemic, this was not related to pre-pandemic decisions but rather dispositional traits (Kneer and Hannikainen, 2021).

### Question I: Utilitarian Preferences and Compliance With Government Guidelines

This research also provided a unique opportunity to investigate whether endorsing certain moral principles predicts real-world behaviours during a pandemic. Recent research has found that individual differences in moral intuitions (such as caring and fairness) predict behaviour compliance versus resistance during the pandemic (Chan, 2021). However, research had yet to consider whether individual differences in endorsements of impartial beneficence and/or instrumental harm predicted behaviour compliance versus resistance during the pandemic. Given that endorsement of these principles could produce different outcomes in the context of the pandemic, we assessed their relationship to behaviours separately. However, we found no evidence that endorsements of instrumental harm or impartial beneficence predicted compliance with government-recommended behaviours in April 2020 or later into the pandemic (September–October 2020). These findings may suggest that the hypothetical nature of sacrificial moral dilemmas and abstract format of the OUS limit the extent to which they apply to related real-world decisions. This is supported by existing research showing the discrepancy between moral judgements and moral behaviours (Patil et al., 2014; Francis et al., 2016). However, it is worth noting that these measures were originally developed to better understand the foundation of human moral cognition, not to predict real-life decisions (e.g., Christensen and Gomila, 2012).

### Limitations and Future Directions

Many of the behaviours investigated in the present study do not involve a trade-off that pits utilitarian- against deontological reasoning. For example, there may be multiple motives for avoiding close contact with someone: self-interested motives, prosocial motives, or rule-based motives driven by authority. Further research during the pandemic could use examples of behaviours that involve a clear trade-off between moral principles.

Additionally, here we measure behaviour compliance using self-report, which suffers from various biases limiting the extent to which we can make claims about real-world behaviours. However, in recent research, members of the public did not under-report their non-compliance with government-recommended behaviours and this provides support for the validity of our measure (Larsen et al., 2020). In the present study, average engagement in government-recommended behaviours was high in both the first (April 2020) and second (September–October 2020) COVID-19 timepoints. Arguably, this could be as a result of the conceptual similarity between the behavioural compliance and moralisation measures used in the present study. However, we know from existing research that there are differential behavioural effects of moralisation on compliant versus non-compliant individuals (e.g., Mulder et al., 2015). As such, future research should attempt to dissociate these by considering whether moralisation predicts behaviour in less compliant groups and by collecting data after the pandemic, when moralisation is likely to be lessened or diminished in some cases.

Given the longitudinal (pre-post) design adopted in this study, our potential pool of participants was constrained. While this longitudinal design enabled us to make inferences about how the pandemic has affected decision-making *within* individuals over time, a larger sample size would have increased the power and subsequent reliability of the present results. This is something future research should consider if another similar event occurs. Importantly, given the sample size of the present study, we could not investigate moralisation and behavioural compliance by country (or region). Although the majority of participants were in lockdowns at timepoint 2 and not in lockdown at timepoint 3, cross-country or cross-regional comparisons should be considered in future research given variations in the type and severity of recommendations and restrictions worldwide. In addition, while we anticipated the strength of moralisation to change as a result of the pandemic, the present study design meant that we did not collect a baseline measure of moralisation and so were unable to account for issues that may have already been moralised prior to the pandemic along with individual differences in strength of moralisation.

In the present study, having close experience of COVID-19 (individuals who reported themselves or a loved one having COVID-19) was not associated with moralisation of pandemic-related behaviours. This may seem somewhat surprising given evidence that the more direct the experience of a disaster, the more prosocial behaviours are displayed (e.g., Kaniasty and Norris, 1995; Rao et al., 2011; Frazier et al., 2013). However, in a recent study, prosocial tendencies have been found to increase with *severity* of the epidemic (Ye et al., 2020). In the present study, we only measured direct experience of COVID-19 rather than the *severity* of the symptoms and outcomes of the disease. Further research should assess these additional factors and their role in moralisation.

### Conclusion

Given that many of the challenges prompted by the COVID-19 pandemic have moral relevance, the present research investigated whether the pandemic is influencing moral judgements and whether moralisation of government advice predicts engagement in recommended behaviours. Overall, we do not find evidence that utilitarian moral judgements within the same individuals have changed during the pandemic, suggesting that fundamental moral codes and ideals have remained stable across time and context. However, individuals appear to have moralised non-compliant and anti-social behaviours associated with the pandemic such as failing to socially distance themselves. Importantly, the process of assigning moral values to these behaviours positively predicts sustained compliance with government recommendations and this has important implications for public messaging strategies during the COVID-19 pandemic.

## Data Availability Statement

The original contributions presented in the study are publicly available. This data can be found on the OSF here: https://osf.io/u5a3t/ (doi: 10.17605/OSF.IO/U5A3T).

## Ethics Statement

The studies involving human participants were reviewed and approved by respective committees at the University of Bradford (2019-E747 and 2020-E803) and the University of Reading (2019-013-CM and 2020-044-CM). The patients/participants provided their written informed consent to participate in this study.

## Author Contributions

Both authors listed have made a substantial, direct, and intellectual contribution to the work, and approved it for publication.

## Conflict of Interest

The authors declare that the research was conducted in the absence of any commercial or financial relationships that could be construed as a potential conflict of interest.

## Publisher’s Note

All claims expressed in this article are solely those of the authors and do not necessarily represent those of their affiliated organizations, or those of the publisher, the editors and the reviewers. Any product that may be evaluated in this article, or claim that may be made by its manufacturer, is not guaranteed or endorsed by the publisher.

## Footnotes

1 The purpose of the original study was to identify moral dilemmas that produced consistent responding (utilitarian or non-utilitarian decisions) within pairs of dilemmas that exhibited similar moral principles (e.g., personal force, intentionality, number of lives saved, etc.). For this study, we selected a threshold of 75% within-subject consistency to correspond to a well-matching dilemma pair. Allowing our 95% confidence interval to extend between 66.5 and 83.5%, where 95% CI = effect size ± 1.96 × standard error (SE) and SE = √[effect size × (1-effect size)/sample size (N)], a sample size of 100 participants was sufficient to identify “consistent” dilemma pairs.

2 Note that two participants were resident in Hungary where localised recommendations rather than localised lockdowns were in place.

3
https://osf.io/n7uts/

4
https://gorilla.sc/

5
https://app.gorilla.sc/openmaterials/250269

6
https://osf.io/ez43n

7 Note that the preregistration states that *t*-tests will be performed. However, this was prior to the third data collection point and as such analyses were extended to ANOVA.
