# Supplementary material for: Moral Decision-Making During COVID-19: Moral Judgements, Moralisation, and Everyday Behaviour
Source: Front Psychol. 2022 Feb 4;12:769177. doi: 10.3389/fpsyg.2021.769177 (PMC8854988; doi:10.3389/fpsyg.2021.769177)
Supplement: Supplementary file 1 [file Data_Sheet_1.pdf]

Supplementary Material

1 Supplementary Information

1.1 Measures: Additional information

In the baseline study, participants responded to 34 moral dilemmas taken or adapted from an existing set of personal moral dilemmas (Christensen et al, 2014). In the COVID-19 studies (timepoints 2 and 3), participants responded to (14) of these dilemmas again. These materials can be found on the project's OSF page: https://osf.io/u5a3t/.

1.2 COVID-19 personal experience checks

To check for associations between personal experience of COVID-19 (confirmed or suspected infection of a participant or someone close to them) and moralisation of behaviours or engagement in government-recommended behaviours, point-biserial correlations were conducted at timepoint 2 (April 2020) and timepoint 3 (September-October 2020). Data were dummy coded: no personal infection or infection of someone close was coded as 0 and a confirmed or suspected personal infection or infection of someone close was coded as 1.

1.3 Self-reported trust in government

To check for associations between self-reported trust in government and moralisation of behaviours and also trust in government and engagement in government-recommended behaviours, correlations were conducted based on self-reported trust at the start of the lockdown and also during the easing of lockdown. Trust scores were calculated by summing responses to three questions phrased retrospectively regarding the start of the lockdown (α = .84) and separately for three questions about the easing of lockdown (α = .87). All questions were administered at timepoint 3.

Participants' self-reported trust in government recommendations was higher at the beginning of the first national lockdown in the UK (37%) compared to during the ease of lockdown (20.6%). Additionally, 41.1% of participants agreed with the statement that government decisions at the beginning of lockdown prioritised public health and safety while 13.7% agreed that government decisions to ease lockdown prioritise public health and safety. Finally, 46.6% of participants agreed that government guidelines at the beginning of lockdown aligned with advice from health experts and this decreased to 28.8% during the easing of lockdown.

1.4 Moralisation of behaviour during COVID-19 (MB-CV19) PCA (Timepoint 2)

A principal component analysis (PCA) with an oblique rotation (Promax) was performed to allow the moralisation items to correlate with each other and to reduce the set of measured items to a smaller set of components (Field, 2013). In initial assessment of the correlation between scale items, no correlation coefficients were greater than 0.9 and for all items, the majority were significantly correlated (*ps* <.05). As such, initial criteria were satisfied for PCA analysis (Field, 2013). In the study preregistration (https://osf.io/ez43n) we specified retaining components with eigenvalues over 1.0. The Kaiser-Meyer-Olkin measure verified the sampling adequacy for the analysis (KMO = .82). Bartlett's test of sphericity ($X^2(190) = 780.89$, $p < .001$) indicated that correlations between items were large enough for PCA (Field, 2013). Firstly, and according to Kaiser's criterion, we found 5 components with eigenvalues over 1.0. However, Kaiser's criterion is accurate when there are less

than 30 items and communalities after extraction are greater than 0.7 or when sample size exceeds 250 (Field, 2013). In the present dataset, although we have 20 items (< 30), several communalities are less than 0.7 and so this criterion may not be accurate. A scree plot was also examined alongside the preregistered criteria specified previously (see Figure 1S), which revealed a drop-off at three components (explaining 53.47% of variance total) and five components (explaining 65.55% of variance total). Factor loadings revealed that several items (17) loaded onto multiple components (> 2) with coefficients greater than .40 (see Table 1S).

Table 1S. Component (factor) loadings for promax PCA on MB-CV19

Item	Item Descriptor	1	2	3	4	5
4	David is organising and hosting a birthday meal at his local pub/bar	**.80**	.22	.21	.15	.17
14	Michelle is coughing without covering her mouth in a public space	**.75**	**.62**	.17	**.44**	**.41**
17	Nicholas is visiting his friend who has a high temperature and persistent cough	**.72**	.26	.34	**.56**	.14
16	Natalie is leaving her home despite feeling unwell	**.71**	**.60**	.23	.34	.34
11	Caroline is taking her children to play in a public playground	**.64**	.20	**.55**	.26	.06
1	Ann throws a house party and invites many friends over	**.64**	.29	.37	**.54**	-.08
13	Stephen is buying toilet roll and other household items in bulk	.12	**.86**	.29	.24	.37
10	Thomas is failing to keep a safe distance from others (2 metres; 6 feet)	**.45**	**.79**	**.42**	**.43**	.24
12	Paul is buying perishable groceries that he does not need/cannot use before they expire	**.63**	**.73**	.28	.35	.35
8	James is travelling to his place of work when he could be working from home	**.55**	**.70**	.43	.37	**.46**
7	Julie is meeting a friend that she does not live with to go for a walk	**.40**	.34	**.83**	**.45**	.26
5	Grace is leaving her home to do exercise more than once a day	.27	.31	**.83**	.36	**.45**

9	Jennifer is travelling to her holiday home to self isolate	.24	**.54**	**.71**	.17	.26
3	Emma is travelling using public transport. She owns a car	**.42**	**.54**	**.57**	.35	**.54**
18	Olivia is visiting her local GP surgery/doctor's practice because she is feeling unwell	.21	.32	.20	**.81**	.26
2	Mark is visiting his 80 year old grandparents	**.49**	.32	.33	**.75**	.16
15	John is not washing his hands regularly	**.44**	**.48**	**.56**	**.65**	.16
6	Daniel sees a friend while out on a walk and greets them by shaking their hand	.17	.21	.38	**.58**	**.45**
19	Matthew is not offering to pick up groceries for a neighbour who is self-isolating after showing symptoms	.28	.36	.30	.25	**.90**
20	Susan is not offering to collect an elderly person's medical prescription	.24	**.49**	.39	.34	**.80**

*Note: One item (3) did not load substantially on to any single component (of the 5 extracted).

Figure 1S

Scree Plot

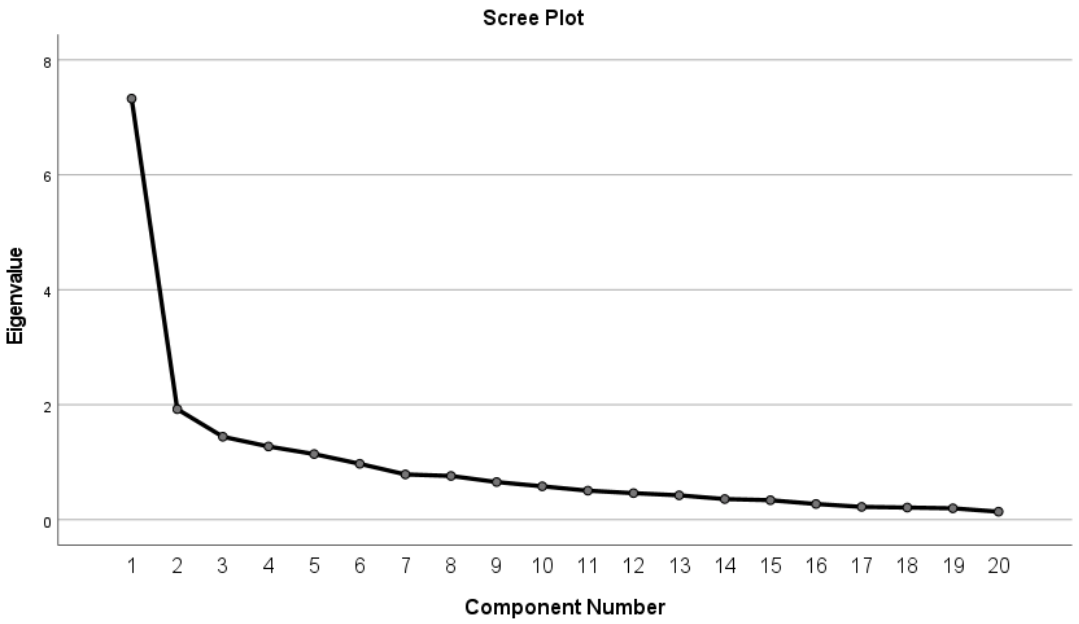

When examining loadings for meaningfulness, the five components were somewhat interpretable (see theoretical interpretation below). *However*, given that many items loaded onto multiple components and that high inter-item correlations were present, a total score for the MB-CV19 was also calculated and used in analyses presented in the manuscript.

The MB-CV19 scores showed good reliability. The overall mean score had an internal consistency of $\alpha = .89$ and the five components had internal consistency scores as follows: Factor 1 $\alpha = .81$, Factor 2 $\alpha = .80$, Factor 3 $\alpha = .75$, Factor 4 $\alpha = .70$, Factor 5 $\alpha = .82$.

Each of the five items on Factor 1 involved a form of *unnecessary risk-taking* such that engaging in those actions would do more harm than good: hosting social events for large groups, using public spaces unnecessarily, exposing oneself and others to illness. Factor 2 items involved *selfish behaviour* that failed to consider others: hoarding supplies, failing to physically distance. Items on Factor 3 concerned actions that *benefit one's own wellbeing*: exercising multiple times a day, visiting your holiday home, meeting a friend for a walk. Factor 4 items all involved *health and hygiene* and more specifically, violations of personal hygiene: visiting the most vulnerable, failing to maintain personal hygiene and hygiene distancing standards. Finally, the items on Factor 5 concerned *failure to take opportunities to do good* or direct prosocial action: not offering to pick up groceries or medical supplies for those in need. With a larger sample size, future research should consider verifying the above component loadings prior to further analyses.

1.5 Behaviours PCA (Timepoint 2)

A principal component analysis (PCA) with an oblique rotation (Promax) was performed to allow the behaviour items to correlate with each other and to reduce the set of measured items to a smaller set of components (Field, 2013). In initial assessment of the correlation between scale items, no correlation coefficients were greater than 0.9. However, there were 4 items that did not correlate significantly with 8 or more other items (the majority). As such, the following items were removed from PCA:

-I am touching my eyes, nose, and mouth with unwashed hands
-I am travelling abroad during the pandemic
-I am wearing a face mask
-I am volunteering to support my local community

All remaining items were significantly correlated (*ps* <.05). As such, initial criteria were satisfied for PCA analysis. In the study preregistration (https://osf.io/u5a3t/) we specified retaining components with eigenvalues over 1.0. The Kaiser-Meyer-Olkin measure verified the sampling adequacy for the analysis (KMO = .81). Bartlett's test of sphericity ($X^2(21) = 121.22$, $p < .001$) indicated that correlations between items were large enough for PCA. Only 1 component had eigenvalues over 1 and explained 57.67% total variance and was therefore retained. Note that any participant with incomplete data was not included in PCA (anyone responding with 'NA' to items 3, 5, 6, and 9). However, this analysis was replicated using mean imputation for missing values and the same component was extracted.

Table 2S. Component (factor) loadings for promax PCA on behaviours

Item	Item Descriptor	1
9	I am self-isolating if showing symptoms or if living with someone showing symptoms	**.90**
3	I am staying at home when I am sick or when I have a cold	**.87**
5	I am covering my mouth when I cough	**.79**
8	I am avoiding places where many people gather	**.77**
6	I am avoiding close contact with anyone who is showing symptoms	**.71**
10	I am staying at least 2m away from people when outside of my home	**.68**
1	I am washing my hands for 20 seconds	**.51**

The behaviour scale showed good reliability. The composite score had an internal consistency of α = .85. The component (and overall scale) represents adherence to government-recommended behaviours (with a focus on UK government policy). The 4 items that were removed were either ill-suited to capturing variance (I am travelling abroad during the pandemic) or were rules/recommendations not explicitly enforced in the UK (I am wearing a face mask; Not touching eyes, nose and mouth; volunteering to support local community).

Moral Decision-Making Before and During COVID-19

Table 3S. Descriptive statistics for OUS overall and subscale scores before and during the pandemic

Scores	Baseline<br>M (SD)	COVID-19 (timepoint 2 in April 2020)<br>M (SD)	COVID-19 (timepoint 3 in September2020)<br>M (SD)

Overall OUS	3.54 (0.85)	3.64 (0.88)	3.69 (0.88)
Impartial Beneficence (OUS-IB)	3.66 (1.14)	3.73 (1.16)	3.72 (1.19)
Instrumental Harm (OUS-IH)	3.39 (1.21)	3.52 (1.12)	3.66 (1.21)

1.6 Moralisation and behaviour regression assumptions

The relationship between the predictor (overall MB-CV19 score) and the outcome variable (overall behaviour score) was linear at both timepoints 2 and 3. Analysis of collinearity statistics show that assumptions were met, as Variance Inflation Factor (VIF) scores were below 10, and tolerance scores above 0.2 (VIF(s) = 1.00, tolerance(s) = 1.00) at both timepoints. The values of the residuals were independent (Durbin-Watson$_{t2}$ = 1.98; Durbin-Watson$_{t2}$ = 2.09). Outlier statistics (Standardised Residual, Cook's Distance, and Leverage Value) identified 1 individual case likely to bias the model at timepoint 3.

1.7 Components of moralization and behaviour (Timepoint 2 only)

As overall mean score was used for subsequent analyses in Francis & McNabb (2020), supplementary regression analyses were performed here on the five extracted components and composite behaviour score at timepoint 2 as specified in the preregistration: https://osf.io/ez43n. For Factors 4 and 5 (*health and hygiene* and *failure to take opportunities to do good*), there was not a statistically significant linear relationship with overall behaviour score, (*ps* > .195) and as such, regression analyses were not performed. For Factors 1-3, the relationship between predictors (Factor 1-3) and the outcome variable (overall behaviour score) was linear. Across models, analysis of collinearity statistics show that assumptions were met (VIF = 1.00, tolerance = 1.00). The values of the residuals were independent (Durbin-Watson = 2.04 - 2.08). No outliers were removed.

Moralisation scores for Factor 1 (*unnecessary risk-taking*) were positively correlated with behaviour scores, ($r(81) = .23$, $p = .034$). The model explained 5.5% of the variance in behaviour scores and was statistically significant, ($F(1, 81) = 4.68$, $p = .034$). Moralisation of behaviour (Factor 1) significantly predicted engagement in government recommended behaviours, ($\beta = .11$).

Moralisation scores for Factor 2 (*selfish behaviour*) were positively correlated with behaviour scores, ($r(81) = .24$, $p = .028$). The model explained 5.8% of the variance in behaviour scores and was statistically significant, ($F(1, 81) = 5.01$, $p = .028$). Moralisation of behaviour (Factor 2) significantly predicted engagement in government recommended behaviours, ($\beta = .09$).

Moralisation scores for Factor 3 (*benefit one's own wellbeing*) were positively correlated with behaviour scores, ($r(81) = .43$, $p < .001$). The model explained 18.1% of the variance in behaviour

scores and was significant, (F(1, 81) = 17.85, p < .001). Moralisation of behaviour (Factor 3) significantly predicted engagement in government recommended behaviours, ($\beta = .12$).

Note and as stated above, when examining loadings for meaningfulness, the five components were somewhat interpretable (see theoretical interpretation below). *However*, given that many items loaded on to multiple components and that high inter-item correlations were present, a total score for the MB-CV19 was also calculated and used in analyses presented in the manuscript.

1.8 OUS-total and behaviour regression assumptions

The relationship between the predictor (OUS-Total scores at baseline) and the outcome variable (overall behaviour score) was linear at both timepoints 2 and 3. Analyses of collinearity statistics show that assumptions were met, as VIF scores were below 10, and tolerance scores above 0.2 (VIF(s) = 1.00, tolerance(s) = 1.00). The values of the residuals were independent (Durbin-Watson$_{t2}$ = 2.14; Durbin-Watson$_{t3}$ = 2.21). Outlier statistics (Standardised Residual, Cook's Distance, and Leverage Value) identified 1 individual case likely to bias the model at timepoint 2.

1.9 OUS-IH and behaviour regression assumptions

The relationship between the predictor (OUS-IH scores at baseline) and the outcome variable (overall behaviour score) was linear at both timepoints 2 and 3. Analyses of collinearity statistics show that assumptions were met, as VIF scores were below 10, and tolerance scores above 0.2 (VIF(s) = 1.00, tolerance(s) = 1.00). The values of the residuals were independent (Durbin-Watson$_{t2}$ = 2.18; Durbin-Watson$_{t3}$ = 2.28). Outlier statistics (Standardised Residual, Cook's Distance, and Leverage Value) identified 1 individual case likely to bias the model at timepoint 2 and 1 individual case likely to bias the model at timepoint 3.

1.10 Moral decision-making: Previous repeated measures data

The following figure shows data from a previously registered study: https://osf.io/kq325. In study a) participants received different moral dilemmas at time 1 and time 2 (these were matched for moral principles). In study b) a separate set of participants received the same moral dilemmas at time 1 and time 2.

Figure 2S

Utilitarian proportions given in response to moral dilemmas presented at two time points

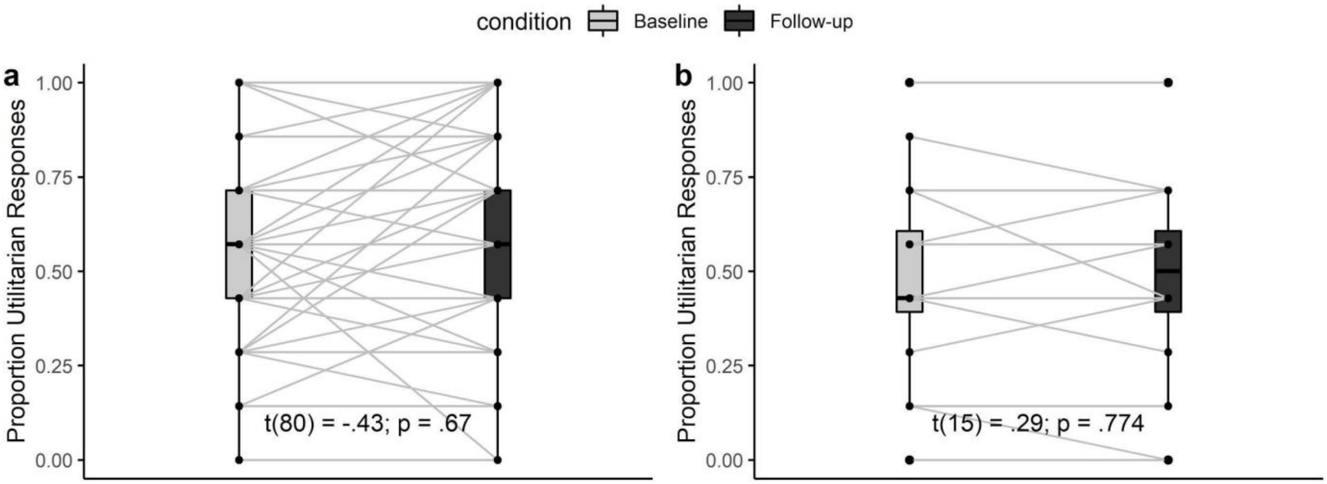

Note. Both studies were within-subjects designs carried out in a student population with 2-4 weeks between time 1 and time 2. Further details about these studies can be found on the OSF: https://osf.io/xvbrf/.
